# TGF-β-induced PLEK2 promotes metastasis and chemoresistance in oesophageal squamous cell carcinoma by regulating LCN2

**DOI:** 10.1038/s41419-021-04155-z

**Published:** 2021-10-02

**Authors:** Feng Wang, Chaoqi Zhang, Hong Cheng, Chengming Liu, Zhiliang Lu, Sufei Zheng, Sihui Wang, Nan Sun, Jie He

**Affiliations:** 1 Department of Thoracic Surgery, National Cancer Center/National Clinical Research Center for Cancer/Cancer Hospital, Chinese Academy of Medical Sciences and Peking Union Medical College, Beijing, China; 2 National Cancer Center/National Clinical Research Center for Cancer/Cancer Hospital, Chinese Academy of Medical Sciences and Peking Union Medical College, Beijing,, China

**Keywords:** Cancer, Molecular biology

## Abstract

Oesophageal squamous cell carcinoma (ESCC) has a relatively unfavourable prognosis due to metastasis and chemoresistance. Our previous research established a comprehensive ESCC database (GSE53625). After analysing data from TCGA database and GSE53625, we found that PLEK2 predicted poor prognosis in ESCC. Moreover, PLEK2 expression was also related to the overall survival of ESCC patients undergoing chemotherapy. Repression of PLEK2 decreased the proliferation, migration, invasion and chemoresistance of ESCC cells in vitro and decreased tumorigenicity and distant metastasis in vivo. Mechanistically, luciferase reporter assay and chromatin immunoprecipitation assay suggested that TGF-β stimulated the process that Smad2/3 binds to the promoter sequences of PLEK2 and induced its expression. RNA-seq suggested LCN2 might a key molecular regulated by PLEK2. LCN2 overexpression in PLEK2 knockdown ESCC cells reversed the effects of decreased migration and invasion. In addition, TGF-β induced the expression of LCN2, but the effect disappeared when PLEK2 was knockdown. Moreover, AKT was phosphorylated in all regulatory processes. This study detected the major role of PLEK2 in driving metastasis and chemoresistance in ESCC by regulating LCN2, which indicates the potential use of PLEK2 as a biomarker to predict prognosis and as a therapeutic target for ESCC.

## Introduction

Oesophageal cancer (EC) is a leading cause of death induced by malignant tumours [1]. As a major histological subtype of EC, oesophageal squamous cell carcinoma (ESCC) is highly prevalent in China [2]. The prognosis of ESCC patients is unsatisfactory mainly because of late presentation, tendency to recur and metastasize, and acquired chemoresistance. Our research group has advantages of mass clinical data and samples, as well as well-built databases. Several biomarkers have been found to indicate the prognosis of ESCC [3, 4]. Thus, we conducted a comparative analysis of both TCGA and our own ESCC database (GSE53625) and found that *Pleckstrin 2 (PLEK2)* has the greatest potential to indicate prognosis.

PLEK2 was first reported to be homologous to Pleckstrin 1. It was reported to be 39% analogous and 65% homologous to the original pleckstrin [5]. PLEK2 has been shown to be expressed in different types of tissues, while Pleckstrin 1 is limited to several immune cells [6]. The modular domains of PLEK2 can bind to membrane-associated phosphatidylinositol [7], which is associated with actin rearrangement in a PI3K-dependent manner [8]. Naume et al. demonstrated that metastasis to bone marrow in luminal A-type breast cancer was highly associated with PLEK2 expression [9]. It might be a marker to distinguish melanoma patients from healthy people in CD45^-^ populations [10]. What’s more, PLEK2 is highly expressed in gallbladder cancer (GBC) and NSCLC [11, 12]. After detecting the former RNA sequence result of TGF-β-induced differential genes in ESCC cell lines, PLEK2 was to be upregulated. In the beginning period of SCC metastasis, TGF-β1-induced EMT plays a critical role [13]. Cancer-associated fibroblasts (CAFs), typical stromal cells, participates in SCC tumour progression by secreting TGF-β1. The high secretion of TGF-β1 activates Yap1 and MMPs, thus accelerating matrix stiffness and eventually facilitating OSCC invasion [14, 15]. As a small-secreted glycoprotein, Lipocalin 2 (LCN2) acts as an innate immune protein and regulates inflammatory conditions [16] and is involved in various types of malignant tumours [17]. High expression of LCN2 is reported to predict poor prognosis in human primary breast cancer, and breast cancer expressing LCN2 indicates a poorly differentiated phenotype [18]. Despite the abovementioned findings, the clinical relevance of PLEK2 and its molecular mechanism in ESCC have not been reported.

In this study, PLEK2 was found to have increased expression in ESCC and the ability to predict poor overall survival (OS). Repression of PLEK2 decreased the proliferation, migration, invasion and chemoresistance of ESCC cells in vitro and decreased tumorigenicity and distant metastasis in vivo. On the other hand, elevating PLEK2 levels had the opposite effects on ESCC cells. TGF-β can increase the expression of PLEK2 by stimulating Smad2/3, which can directly bind to the promoter sequence of PLEK2. Analyses of transcriptome profiling suggested that LCN2 is downstream of PLEK2. Overexpression of LCN2 in ESCC cells with stable PLEK2 knockdown reversed the decrease in migration and invasion but did not decrease the proliferation of ESCC cells. Moreover, TGF-β increased the expression of LCN2, but the effect disappeared when PLEK2 was knocked down. We also found that the AKT pathway is involved in all regulatory processes. This article indicates the potential of PLEK2 as a biomarker to predict prognosis and as a possible target for clinical therapy against ESCC.

## Materials and methods

### Characteristics of patients and clinical samples

ESCC tissues and paired adjacent nontumour tissues were separated from patients recruited from the National Cancer Center. The tissues were used to develop a tissue array. The use of these tissues was approved by the Institutional Review Board and the Research Committee of the National Cancer Center, and all participants signed informed consent forms. None of the patients received radiotherapy or chemotherapy before the operation. The detailed characteristics of these patients are shown in Supplementary Table 1. Tumour tissues were got from ESCC patients enroled in GSE53625 who received chemotherapy to detect the level of PLEK2 and the characteristics of these patients are shown in Supplementary Table 2.

### Animal experiments

Balb/c null (female, 4–6 weeks) were used in the animal experiments. After the mice were purchased and maintained for at least 1 week, they have conducted the experiments. All the mice were divided randomly into two groups. All the procedures were approved by the Institutional Animal Care and Use Committee at National Cancer Centre. A total of 2 × 10^6^ ESCC cells were injected subcutaneously for initiating tumour and there were 6 mice in each group. Tumour volume was calculated as follows: tumour volume = length × width^2^/2. Tumour volume was calculated every day by investigators who were blinded to the group allocation and the growth curve was drawn according to the collected data. What’s more, 1 × 10^6^ ESCC cells were injected per mouse to conduct a tail vein injection model for lung colonization assay. There were 5 mice in each group. To detect the metastasis of ESCC cells, 5 × 10^6^ ESCC cells were injected to the foot pad of each mouse(3 mice in each group) and popliteal lymph node were separated after 2 months.

### Regents and antibodies

All regents including Cisplatin (Sigma, p4394) and Recombinant Human TGF-β1 (R&D, 240-B/CF) were obtained from commercial sources. The following antibodies were used: anti-PLEK2 (1:200 for IHC, 1:500 for Western blot, Proteintech, 11685-1-AP), anti-α-Tubulin(1:5000, Sigma, T9026), anti-Akt (1:1000, CST, #9272), anti-p-Akt Antibody(Ser 473)(1:1000, CST, #4060), anti-Erk (1:1000, CST, #4695), anti-p-Erk (1:500, CST, #4370), anti-Claudin-1 (1:500, CST, #13255), anti-Cyclin B1 (1:1000, CST, #4118), anti-Cyclin E2 (1:1000, CST, #4132), anti-Bax (1:1000, CST, #14796), anti-Bcl-2 (1:!000, CST, #3498), anti-PARP (1:1000, CST, #9532), anti-Cleaved-PARP (1:1000, CST, #9548), anti-Caspase 3 (1:1000, CST, #9662).

### Cell culture

The ESCC cell lines KYSE-30, KYSE-450, KYSE-70, KYSE-150, and KYSE-180 and the immortalized human oesophageal cell line Het-1A were purchased from the Cell Bank of Peking Union Medical College (Beijing, China). Cell lines mentioned above were recently authenticated and tested for mycoplasma contamination. The ESCC cell lines were cultured in RPMI 1640 containing 10% foetal bovine serum (FBS). The cells were incubated in an incubator with 5% CO_2_ at 37 °C.

### Colony formation

Five hundred cells were inoculated in 6-well plates. The inoculated cells were then cultured in medium containing 10% FBS for at least fourteen days. Colonies were fixed with 4% paraformaldehyde for at least 30 min at room temperature and then stained with crystal violet for at least 10 min at room temperature. The number of colonies of each group was counted and statistically analysed.

### Cell counting kit-8 (CCK-8) assay

A total of 3 × 10^3^ cells in each plate were incubated under the conditions mentioned in the cell culture section in 96-well plates. At 0 h, 24 h, 48 h, 72 h and 96 h, 10 μl CCK-8 solution mixed with 90 μl RPMI 1640 medium was added to each plate and incubated for 2 h at 37 °C before detection.

### Cell migration and invasion assays

Different transwell chambers were chosen to detect migration and invasion respectively. The treated ESCC cells were digested and resuspended in serum-free medium. In the upper chamber, 5 × 10^4^ cells were inoculated gently. Moreover, the lower chamber was filled with RPMI-1640 medium with 20% FBS. Twenty-four hours later, the migrated and invaded cells were stained and counted in six different fields.

### Wound healing assay

Cells treated with different methods were incubated into plates. After being treated for 24 h with mitocycin C, a linear wound was made in each plate. Then PBS was used to wash the detached cells from the plates. Then serum-free medium was added into the plates. After been incubated 12 h, 24 h, and 36 h later, the wounds of different plates were observed and estimated.

### Quantitative real-time polymerase chain reaction (qRT-PCR)

TRIzol Reagent was used to extract RNA following the manufacturer’s protocol. A RevertAid First Strand cDNA Synthesis Kit was chosen to conduct the reverse transcription experiment. First-strand cDNA was generated during the procedure. To detect the expression of several genes, qRT-PCR following the SYBR protocol was carried out on a 7900 Real-Time PCR System by using SYBR^TM^ Select Master Mix. Primers sequences are shown in Supplementary Table 3.

### Western blot

RIPA lysis reagent mixed with protease inhibitor was used to extract the proteins from cells, and the concentration of cell lysates was detected by a BCA assay. Equal quantities of proteins were separated by 10% sulfate-polyacrylamide gel electrophoresis (SDS-PAGE), and then the proteins were transferred to PVDF membranes. Subsequently, nonspecific antigens on the membranes were blocked by incubating the membranes in 5% skim milk. Several primary antibodies were incubated with the membranes at 4 °C overnight. The corresponding HRP-conjugated secondary antibodies were then applied to the membranes and incubated for at least two hours. The signals of each washed membrane was detected by electrochemiluminescence.

### Chromatin Immunoprecipitation (ChIP)

JASPER website was used to predict the binding motifs of Smad2/3. The predicted sequences are presented in Supplementary Table 4. ChIP was performed following the instructions of a commercial CST kit called the Simple ChIP Enzymatic Chromatin Immunoprecipitation Kit. The outcome was detected by RT-qPCR. The primer sequences specific for the promoter region of PLEK2 are displayed in Supplementary Table 5.

### Luciferase reporter assay

The potential promoter sequences were designed and synthesized by Beijing Syngentech Corporation (Beijing, China). KYSE-30 cells were co-transfected with reporter plasmids containing the potential promoters of PLEK2 and a Renilla luciferase vector. All the above-mentioned cells were cultured in complete medium with 10 μM TGF-β. After the cells were cultured for at least 48 h, the firefly luciferase activity and Renilla luciferase activity of cells were detected by using a Dual-Luciferase^®^ Reporter Assay System (Promega).

### Stable overexpression or knockdown of PLEK2 in ESCC cell lines

The PLEK2 shRNA expression vector and scrambled shRNA nontarget control were obtained from GenePharma. Sequences of shRNA directed against PLEK2 were sh-1: GGATCCAGCTTTCCTGCAT and sh2: GGCTCATCTCCAACAGCTTCA. PLEK2 lentiviral overexpression vector and empty vector were purchased from Beijing Syngentech Corporation (Beijing, China).

### Cell cycle detection

An APC BrdU Flow Kit (BD, 552598) was used to detect the cell cycle. After synchronizing the cell cycle for 24 h, the cells were resuspended and fixed with 75% ethanol for at least 4 h. Finally, the cells were stained with PI, which is the dye used to detect the cell cycle population by using flow cytometry. All the data were analysed by Flowjo X.

### Cell apoptosis detection

An Annexin V Apoptosis Detection Kit APC (KeyGEN Biotech, KGA1021) was used to detect the percentage of apoptotic cells. When the cells confluence reached 70–80%, cells were digested and resuspended, followed by staining with 10 *µ*l Annexin V-APC and PI for 20 min in the dark. The percentage of apoptotic cells was measured by a BD LSR II flow cytometer, and the results were analysed by FlowJo X. To detect the reaction of ESCC cells to DDP, the cells were treated with 30 μM DDP for at least 24 h and then collected to detect.

### Immunohistochemistry (IHC)

Slides were heated at 65 °C for half an hour and then heated in a microwave oven for antigen retrieval. After the elimination of endogenous peroxidase activity and the block of nonspecific binding, slides were incubated with a monoclonal antibody against PLEK2 overnight at 4 °C. Subsequently, the slides were incubated with horseradish peroxide avidin biotin complex for another 1 h and the reaction products were visualized with a diaminobenzidine (DAB) chromogen solution. Evaluation of PLEK2 expression was performed by at least two pathologists who did not have knowledge of the patients’ diagnoses.

### Statistical analysis

All experiments were conducted at least three times, and each value came from at least three biological repeats. SPSS 13.0 was used to analyse the gained value and obtain *P* values. GraphPad 7.0 was used to illustrate the graphs based on the analysed results. The results of the cell experiments were presented as the mean ± SD from three independent experiments, and the differences among the two groups were analyzed by an independent samples Student’s *t* test. The Kruskal–Wallis non-parametric test was used to analyse unpaired data. There were three levels of statistical significance (*P < 0.05; **P < 0.01; ***P < 0.001).

## Results

### PLEK2 is overexpressed and associated with poor prognosis in ESCC

**Fig. 1:**
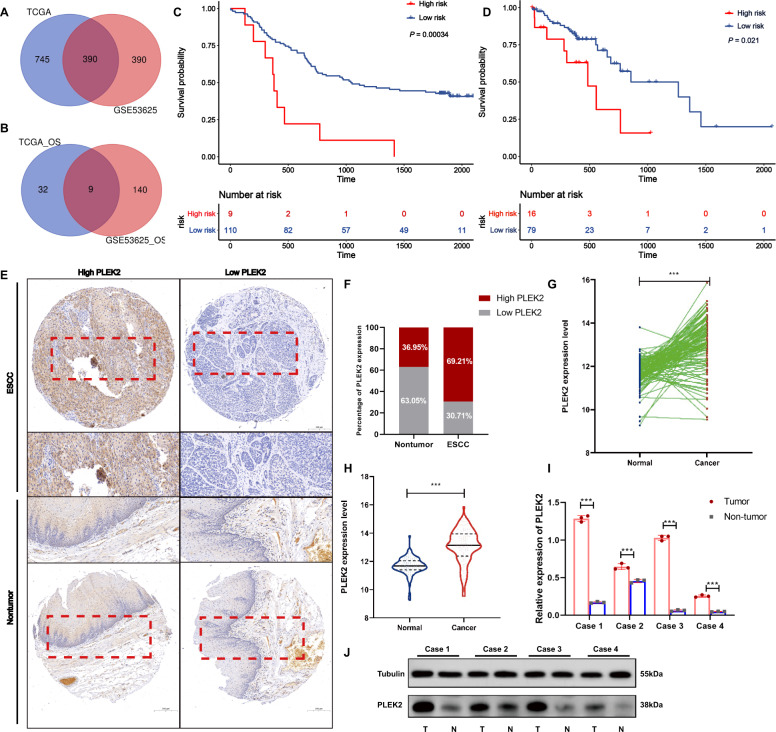
PLEK2 is overexpressed in ESCC and associated with prognosis. **A** Venn diagram of common differentially expressed genes in the TCGA and GSSE53625; **B** Venn diagram of common genes which were related to OS in the TCGA and GSSE53625; **C** Kaplan–Meier plots of OS of patients with ESCC in GSE53625, *P* = 0.00034; **D** Kaplan–Meier plots of OS of patients with ESCC in TCGA database, *P* = 0.021; **E** PLEK2 expression in a human ESCC tissue microarray. Representative microscopic images of benign and ESCC tissues stained with an antihuman PLEK2 antibody; **F** Bar chart summary of the distribution of PLEK2 expression levels in nontumor versus ESCC by IHC (*n* = 100 total cases; *P < 0.05); **G** The expression of PLEK2 in tumour tissues and nontumor tissues in the GSE53625 database when the data were not paired, ***P < 0.001; **H** The expression of PLEK2 in tumour tissues and nontumor tissues in the GSE53625 database when the data were paired, ***P < 0.001; **I** Relative expression of PLEK2 in fresh tumour tissues and adjacent normal tissues from four ESCC patients detected by Western Blot. ***P < 0.001; **J** Expression of PLEK2 in fresh tumour tissues and adjacent normal tissues from four ESCC patients detected by Western Blot. Tubulin was used as loading control.

By analysing the ESCC data from TCGA database and the database of our own lab which named GSE53625, we found that 1670 genes exist with different expression levels in ESCC tissues and paired nontumour tissues (|Log Fc | >1, *P* < 0.05). Additionally, we detected 12257 differentially expressed genes in the GSE53625 database (|Log Fc | >1, *P* < 0.0001). There were 390 common differentially expressed genes in the two datasets (Fig. 1A). Among them, 32 genes were related to the OS of ESCC patients in TCGA database, and 140 genes were related to OS in the GSE53625 database. Eventually, 9 genes were selected as the genes common to the two datasets (Fig. 1B). PLEK2 was identified to have maximal HR among all 9 differentially expressed genes. Then, we evaluated the relationship between PLEK2 and the OS of ESCC patients in TCGA and GSE53625 databases. The results showed that higher expression of PLEK2 indicated shorter OS in ESCC patients of TCGA and GSE53625 databases with *P* values of 0.00034 and 0.021, respectively (Fig. 1C, D). However, the expression of PLEK2 was shown to have no correlation with PFS of ESCC patients (Supplementary Fig. S1). The IHC results showed the expression level of PLEK2 in the ESCC tissue microarray (Fig. 1E), and it is obvious that PLEK2 was more highly expressed in ESCC tissues than in nontumour tissues (Fig. 1F). The expression of PLEK2 and data in the GSE53625 database showed similar results when either the tissue was not paired or paired (Fig. 1G, H). The detection of PLEK2 expression in paired fresh tumour tissues and adjacent normal tissues from four ESCC patients who underwent surgery showed that PLEK2 expression was higher in tumour tissues than in paired adjacent nontumour tissues (Fig. 1I, J). All the above results indicate that PLEK2 is overexpressed in ESCC and predicts poor prognosis of ESCC patients.

### PLEK2 knockdown inhibits ESCC cell proliferation through redistribution of the cell cycle

**Fig. 2:**
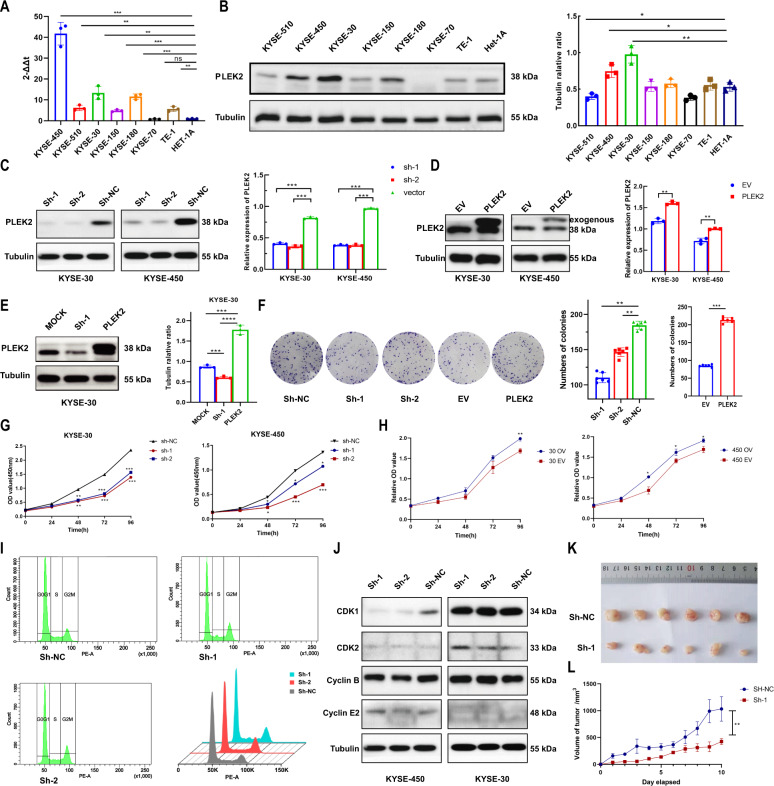
Silence of PLEK2 inhibited proliferation of ESCC cells in vivo and vitro. **A** Expression of PLEK2 in ESCC cell lines detected by qPCR; **B** Western blot analysis of level of PLEK2 in several ESCC cell lines; **C** Silence of PLEK2 expression in KYSE-30 and KYSE-450. Western Blot was used to confirm the knockdown efficiency; **D** Overexpression of PLEK2 expression in KYSE-30 and KYSE-450 Western Blot was used to confirm the overexpression efficiency; **E** Reverse effect of PLEK2 overexpression plasmid on PLEK2 stably knockdown KYSE-30 and KYSE-450; **F** The colony generated by KYSE-30 with or without PLEK2 stably knockdown, as well as KYSE-30 with or without PLEK2 stably overexpressed. **G** Proliferation ability of stably knockdown PLEK2 in KYSE-30 and KYSE-450 detected by CCK-8 assay; **H** Proliferation ability of stably overexpression PLEK2 in KYSE-30 and KYSE-450 detected by CCK-8 assay; **I** Flow cytometry diagrams of cell cycle of both KYSE-30 with or without PLEK2 stably knockdown. **J** Western blot analysis for the expression of CDK1, CDK2, Cyclin B, and Cyclin E2, which related to cell cycle. Tubulin was used as loading control; **K** Representative xenograft tumours derived from mice subcutaneously injected with KYSE-30 cells with or without PLEK2 stably knockdown. (*n* = 6 in each group). **L** The growth curve of tumours in two groups (*n* = 6 in each group). *P < 0.01; ***P < 0.01; ***P < 0.001.

To verify the findings from the database and clinical samples, PLEK2 expression in normal oesophageal epithelial cell lines and different ESCC cell lines was monitored by using relative quantification methods, such as Western blot and qPCR. ESCC cell lines expressed higher mRNA and protein levels of PLEK2 (Fig. 2A, B), and the top two cell lines were KYSE-450 and KYSE-30. Thus we conducted knockdown of PLEK2 expression in the two cell lines and the knockdown efficiencies were both above 50% (Fig. 2C). We also conducted the overexpression of PLEK2 in two cell lines and found exogenous PLEK2 expression due to the use of plasmid with 3×FLAG(Fig. 2D). To detect whether the knockdown site was appropriate, the overexpression plasmid was used to revert the knockdown effect (Fig. 2E). After detecting the biological function of conducted cell lines, it was found that PLEK2-knockdown cells had a poorer colony formation capacity, while PLEK2-overexpressing cells showed a better colony formation capacity (Fig. 2F). Knock down the expression of PLEK2 inhibited cell proliferation of KYSE-30 and KYSE-450 cells (Fig. 2G). Conversely, overexpression of PLEK2 in the two cell lines promoted the proliferation of ESCC cell lines (Fig. 2H). The overall reason for the inhibition of PLEK2 knockdown may be associated with cell cycle arrest in the G2/M-phase (Fig. 2I). Cell cycle associated markers were detected by western blot (Fig. 2J), as the cyclin of M cycle, the expression of Cyclin B was increased in KYSE-30 when PLEK2 was knockdown. However, Cyclin E2 and CDK2, which were associated with G1 cycle, showed no significant expression difference in cells with PLEK2 knockdown. Moreover, when PLEK2 was knocked down in the KYSE-30 cell line, its tumorigenic ability was significantly decreased when the cells were subcutaneously xenografted (Fig. 2K, L).

### PLEK2 knockdown inhibits ESCC cell metastasis

**Fig. 3:**
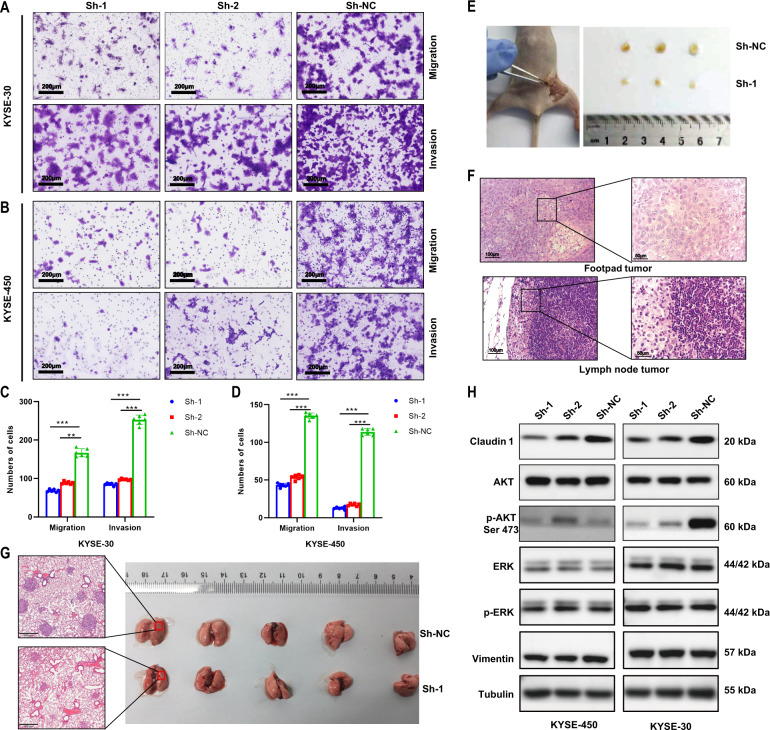
Silence of PLEK2 inhibited metastasis of ESCC cells in vivo and vitro. **A**, **B** The migration and invasion ability of PLEK2 knockdown cells including KYSE-30 and KYSE-450; **C**, **D** Qualification of number of cells that migrated through the membrane or invaded through the membrane; **E** Lymph node metastasis of KYSE-30 with PLEK2 stably knockdown; **F** Representative H&E images of popliteal lymph node; **G** Resected lung sections derived from mice injected via tail vein with KYSE-30 cells with or without PLEK2 stably knockdown; **E** Western blot analysis for the expression of Claudin-1, AKT, p-AKT (ser 473), ERK, p-ERK, and Vimentin. Tubulin was used as loading control.

PLEK2 knockdown inhibited the migration and invasion of ESCC cells. Knockdown of PLEK2 resulted in a critical decrease in the migration and invasion capabilities of cells to pass through an extracellular matrix coating (Fig. 3A, B). Moreover, stable PLEK2 overexpression resulted in the opposite effects (Supplementary Fig. S2A). When the cell cycle was synchronized, similar results were obtained, suggesting that the PLEK2-mediated tendency to migrate and invade ESCC cells was not a misapprehension of the cell-modified tendencies towards proliferation (Supplementary Fig. S2B). To further validate this conclusion, both KYSE-30^PLEK2shRNAs^ cells and ESCC cells transfected with an empty vector were separately injected into the two sides of the footpad of each mouse to generate the lymph node metastasis model. Popliteal lymph nodes were separated 80 days after injection. The group of stable knockdown cell lines had smaller lymph nodes than the control group (Fig. 3E). H&E staining of separated lymph nodes suggested that a large number of tumour cells had metastasized to lymph nodes (Fig. 3F). Likewise, compared with the normal control group, KYSE-30^PLEK2shRNAs^ exhibited a decreased ability to metastasize to the lungs after the cells were injected through the tail vein (Fig. 3G). Histological analysis revealed that the KYSE-30^PLEK2shRNAs^ group had fewer metastatic foci in the lung tissues than the NC group. Western blot analysis detected markers related to tumour metastasis and potential pathways. The results showed that when PLEK2 was stably knocked down, Claudin-1 was significantly decreased, and no change in vimentin expression was detected. The Akt pathway was involved in the knockdown process but not the other pathways, such as the ERK/MERK pathway (Fig. 3H).

### PLEK2 contributes to chemoresistance potential in ESCC

**Fig. 4:**
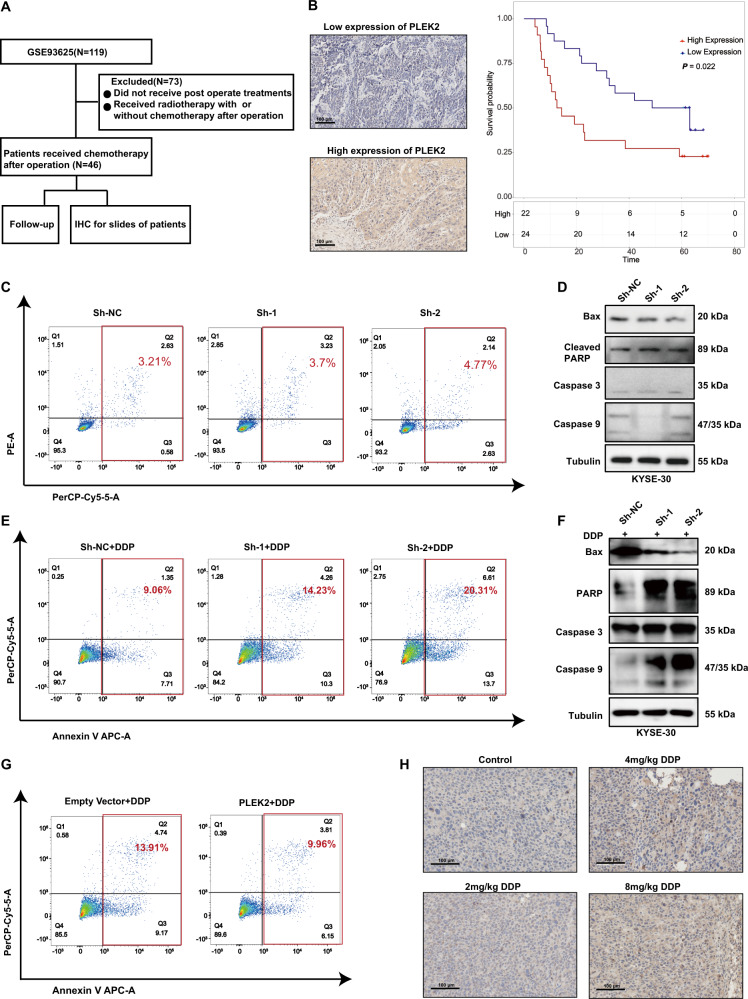
PLEK2 linked to chemo-resistance of ESCC cell lines. **A** The scheme of process of choosing patients; **B** Representative images of high and low expression of PLEK2 (left) and Kaplan-Meier plots of OS of patients who received chemotherapy in the database GSE53625 (right); **C** The apoptosis rate of PLEK2 stably knockdown KYSE-30 cells; **D** Western blot analysis of markers of cell apoptosis including Bax, Cleaved PARP, Caspase 3 and Caspase 9. Tubulin was used as loading control; **E** The apoptosis rate of PLEK2 stably knockdown KYSE-30 cells after the treatment of DDP for 24 h; **F** Western blot analysis of markers of cell apoptosis; **G** The apoptosis rate of KYSE-30 with PLEK2 stably overexpressed after the treatment of DDP for 24 h; **H** Representative images of IHC in KYSE-30 xenograft tumours treated with varying dosage of DDP.

By detecting the level of PLEK2 in tumour tissues from ESCC patients who received chemotherapy (Fig. 4A), we found that high PLEK2 expression linked to the OS of the chosen patients (Fig. 4B). Therefore, the influence of PLEK2 knockdown on chemoresistance was detected. Before chemotherapy, whether PLEK2 knockdown influenced the apoptosis of ESCC cells was detected. There was no difference between the PLEK2-knockdown cell apoptosis analysis results from the NC group cells, thus indicating that PLEK2 influences the proliferation of ESCC cells but not by influencing the apoptosis of cells (Fig. 4C). The expression of markers associated with apoptosis, such as Bax, cleaved PARP, caspase 3, and caspase 9, showed no increase after stable PLEK2 knockdown (Fig. 4D). When the cells were treated with cisplatin at a concentration of 30 μM, the ratio of apoptosis of KYSE-450^PLEK2shRNAs^ and KYSE-30^PLEK2shRNAs^ cells was significantly higher than that of normal control cells (Fig. 4E). Additionally, the expression of Bax was decreased and the expression of cleaved PARP, caspase 9, and caspase 3 was upregulated in both KYSE-450^PLEK2shRNAs^ and KYSE-30^PLEK2shRNAs^ cells (Fig. 4F). In contrast, the PLEK2-overexpressing cells had increased survival after cisplatin treatment; thus, PLEK2 overexpression may induce resistance to cisplatin (also called DDP) (Fig. 4G). Furthermore, we treated mice which were subcutaneously xenografted ESCC cells with dosage of 0 mg/kg, 2 mg/kg, 4 mg/kg, and 8 mg/kg cisplatin. Results of IHC conducted on tumour tissues indicated that the expression of PLEK2 was gradually increased as the dosage increased (Fig. 4H).

### TGF-β induces PLEK2 expression and its potential function in phenotypic alteration

**Fig. 5:**
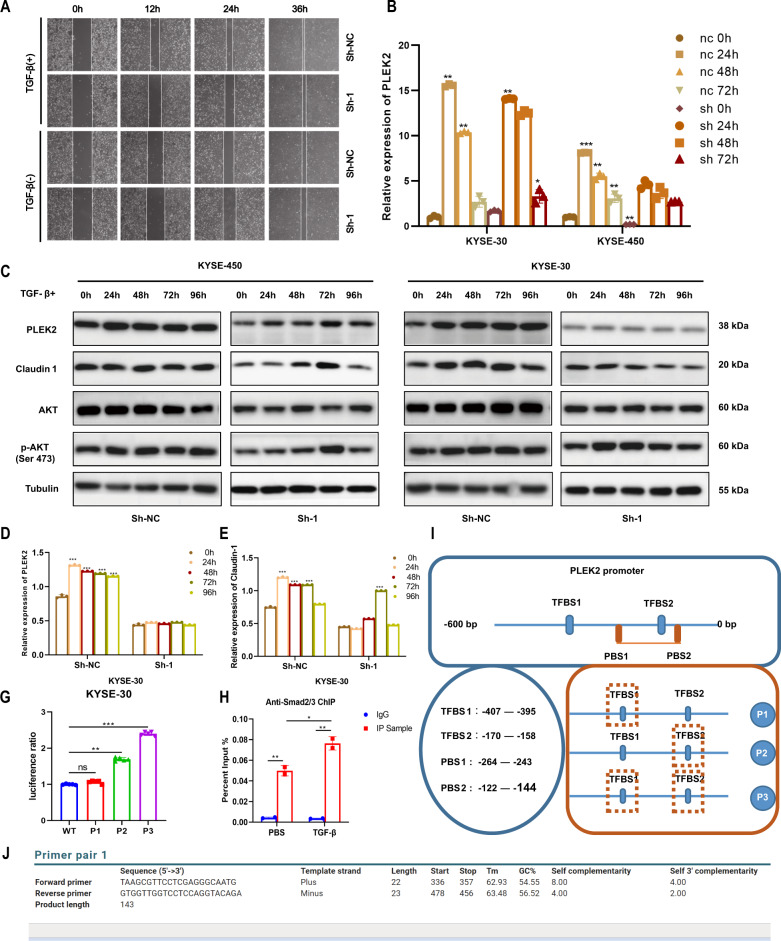
TGF-β induces PLEK2 expression and its potential function. **A** Wounds of KYSE-30 cells with or without PLEK2 stably knockdown treated by TGF-β or PBS; Relative expression of PLEK2 in KYSE-30 with or without PLEK2 stably knockdown when treated by TGF-β in different time points. **C** Western blot analysis of PLEK2, Claudin-1, AKT, and p-AKT (Ser 473). Tubulin was used as control; **D** Histogram of expression of PLEK2 in KYSE-30 with or without PLEK2 stably knockdown when treated by TGF-β in different time points; **E** Histogram of expression of claudin-1 in KYSE-30 with or without PLEK2 stably knockdown when treated by TGF-β in different time points; **G** Luciferase of cells which were transferred into different plasmid containing different promoter regions; **H** ChIP assay showed interaction between Smad2/3 and PLEK2; **I** P1, P2, P3 sites (tested in luciferase assays) nearby PLEK2 gene; TFBS, transcription factor binding site; PBS, Primer Binding Site; **J** the ChIP primers location in PLEK2 promote. *P < 0.01; ***P < 0.01; ***P < 0.001.

Our laboratory has conducted RNA-seq experiment on ESCC cell lines which were treated by TGF-β. PLEK2 was found to be among the up-regulated genes in KYSE-30 and KYSE-180 cell lines with average log_2_^FC^ at 3.12 and 1.7 respectively (Supplementary Fig. S3). PLEK2 knockdown cells showed lower migration capability when treated with TGF-β (Fig. 5A). TGF-β is associated with the expression of PLEK2, and with continuous stimulation by TGF-β at the concentration of 10 μM, the level of PLEK2 was upregulated over time (Fig. 5B). The level of PLEK2 Claudin-1 was upregulated on protein level, especially in KYSE-30 cells. The stimulation of TGF-β in the stable knockdown PLEK2 cells also upregulated PLEK2 and Claudin-1 expression, but the expression level was lower than the expression level in the normal control cells after TGF-β stimulation at the same time point (Fig. 5C–E). We then detected how TGF-β influenced the expression of PLEK2 and its downstream effectors. Three promoter sequences were predicted according to the data in the JASPER database. The promoter sequences were inserted into plasmid, and when the cells were stimulated with TGF-β, the plasmid was transfected with the renewal plasmid. After the detection of the dual luciferase signal, we found that P3 had the highest ratio, suggesting that TGF-β-induced transcription factors may directly bind to the third sequence of PLEK2 and upregulate its expression (Fig. 5G). Later, we used Smad2/3 antibodies to conduct ChIP assay and found increase of enriched PLEK2 promoter sequences in TGF-β treatment cells (Fig. 5H). Briefly, P1, P2, P3 sites which were tested in luciferase assays nearby PLEK2 gene are shown in Fig. 5I. The ChIP primers location in PLEK2 promote is shown in Fig. 5J.

### PLEK2 functions as an oncogene through the regulation of LCN2

**Fig. 6:**
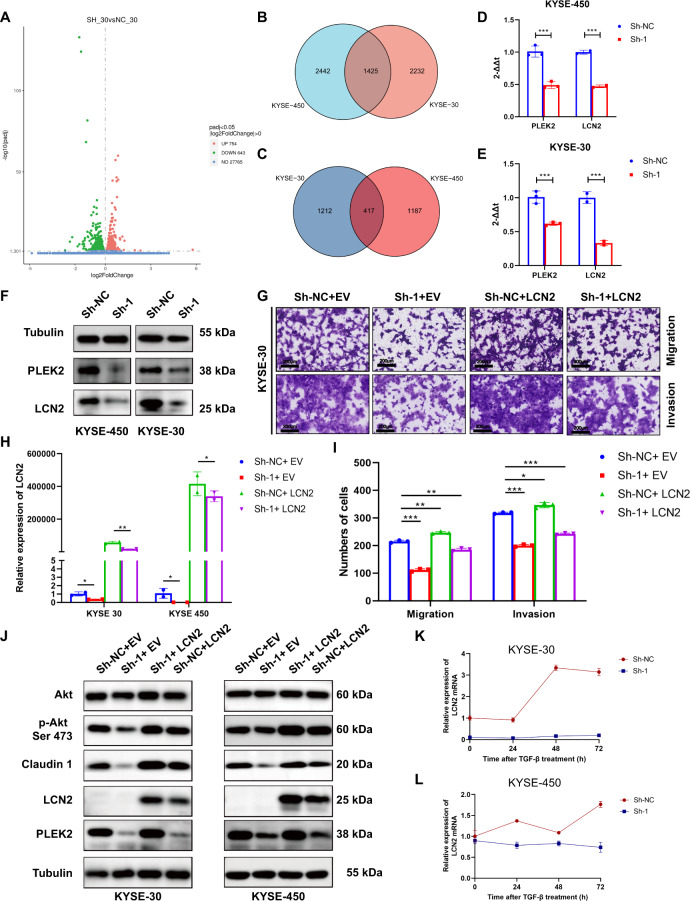
LCN2 is detected to be directly regulated by PLEK2. **A** Volcano plot of differential genes expression after PLEK2 was stably knockdown in KYSE-30 cells. **B** The Venn diagram showed number of differential upregulated genes in PLEK2 stably knockdown cells; **C** The Venn diagram showed number of differential downregulated genes in PLEK2 stably knockdown cells; **D** The expression of PLEK2 and LCN2 in PLEK2 knockdown KYSE-450 cells on the mRNA level; **E** The expression of PLEK2 and LCN2 in PLEK2 knockdown KYSE-30 cells on the mRNA level; **F** The expression of PLEK2 and LCN2 in PLEK2 knockdown KYSE-30 and KYSE-450 tested by western blot. The expression of LCN2 in PLEK2 knockdown cells which treated with TGF-β on the mRNA level; **G** The migration and invasion ability of PLEK2 knockdown cells with or without LCN2 overexpression; **H** Overexpression of LCN2 in PLEK2 knockdown cells and its efficiencies were detected on the mRNA level; **I** Qualification of number of cells that migrated through the membrane or invaded through the membrane; **J** Western blot analysis of PLEK2 Claudin-1, AKT, p-AKT, and LCN2. Tubulin was used as loading control; **K** The expression of LCN2 in PLEK2 knockdown KYSE-30 which treated with TGF-β on the mRNA level; **L** The expression of LCN2 in PLEK2 knockdown KYSE-450 which treated with TGF-β on the mRNA level; *P < 0.01; ***P < 0.01; ***P < 0.001.

To further investigate the inner mechanism by which PLEK2 influences cell proliferation, metastasis, and invasion, RNA-seq data of KYSE-450^PLEK2shRNAs^ and KYSE-30^PLEK2shRNAs^ cells were obtained and analysed (Fig. 6A–C) and LCN2 was found to be the most important downstream marker of PLEK2. The expression of LCN2 was decreased in KYSE-450^PLEK2shRNAs^ and KYSE-30^PLEK2shRNAs^ cells on mRNA level (Fig. 6D, E). The expression of LCN2 was also decreased in KYSE-450^PLEK2shRNAs^ and KYSE-30^PLEK2shRNAs^ cells on protein level (Fig. 6F). To investigate whether the decrease in LCN2 was regulated by PLEK2, a plasmid containing LCN2 overexpression sequences was transfected into KYSE-450^PLEK2shRNAs^ and KYSE-30^PLEK2shRNAs^ cells. The efficiencies of transfection was detected by real-time PCR (Fig. 6H). We found that overexpression LCN2 could reverse the decreased migration and invasion capability of KYSE-30^PLEK2shRNAs^ cells (Fig. 6G, I). No obvious changes were observed in the aspect of proliferation (Supplementary Fig. S4). Moreover, the level of Claudin-1 and the phosphorylation of Akt were also reversed, which is accordance with the functional tests (Fig. 6J). TGF-β also induced the expression of LCN2 in ESCC cells whereas the effect disappeared when PLEK2 was knockdown (Fig. 6K, L). The above findings show strong evidence that LCN2 might be a downstream molecule of PLEK2.

## Discussion

PLEK2 has been poorly studied in malignant tumours. Shen et al. found that PLEK2 promoted the processes of invasion and metastasis in GBC through the EGFR/CCL2 pathway [11]. The upregulation of PLEK2 is linked to the PFS of NSCLC [19], which is related to PLEK2-regulated metastasis and vascular invasion in NSCLC [20]. Previous studies revealed the role of PLEK2 as an oncogene in malignant cancer, and our results are consistent with those of previous studies. However, apart from the abovementioned findings, studies on PLEK2 and malignant tumours are hard to conduct. In this study, we showed for the first time that PLEK2 plays an important role in ESCC. Here, PLEK2 was first shown to have differential expression in tumour tissue and normal oesophageal squamous epithelial tissue, which could be related to the finding that PLEK2 expression predicts a poor prognosis in ESCC. The results also indicated that PLEK2 might be a candidate target to investigate. Moreover, high expression of PLEK2 indicated shorter OS and a poorer chemotherapy response. Chemoresistance is a major reason for recurrence in ESCC patients. Cisplatin is the basis of postoperative therapy [21, 22]. Thus, we detected that lower expression of PLEK2 could result in sensitivity of ESCC cells to cisplatin. According to the results of functional studies, we detected the role of PLEK2 in enabling ESCC cells to be resistant to cisplatin, which would also provide the possibility that PLEK2 can become a marker that predicts the outcome, including chemoresistance and recurrence, of an ESCC patient who receives cisplatin therapy.

TGF-β plays a fundamental role in carcinogenesis and tumour progression. However, most studies have determined that TGF-β has dual effects on this process. Before tumours occur, TGF-β can inhibit normal and precancerous epithelial cells from evolving towards malignant tumours [23]. However, due to the increased gene expression and epigenetic changes in tumour cells, local secretion of TGF-β increased. Thus, TGF-β promotes the occurrence of invasion and metastasis [24, 25]. The effect of TGF-β on ESCC remains controversial. Studies have indicated that TGF-β promotes the progression of ESCC [26, 27]. In our study, we confirmed that TGF-β can significantly increase the levels of PLEK2 and LCN2. Additionally, the levels of both PLEK2 and LCN2 in ESCC are positively related to the progression of migration, invasion and metastasis. The data are consistent with the abovementioned conclusions. Moreover, when PLEK2 was knocked down, the effect of TGF-β on LCN2 nearly disappeared, which strongly predicted that the effect of TGF-β on LCN2 was dependent on PLEK2. The Smad2/3 pathway, usually induced by TGF-β, was predicted to be an upstream molecule of PLEK2. We believe that Smad2/3 is affected as a transcription factor [28, 29] and directly binds to the promoter of PLEK2, thus increasing PLEK2 expression. In the immune environment of ESCC, CAFs are a major source of TGF-β [30], which may be the main reason that PLEK2 is highly expressed in ESCC tumours.

Our results showed that silencing the expression of PLEK2 in ESCC cell lines had a remarkable effect on attenuating the proliferation, migration, invasion, and metastasis of ESCC cells. In contrast, PLEK2 overexpression in KYSE-450 and KYSE-30 cells enormously increased their invasive ability. Multiple analyses of transcriptome sequences showed that LCN2 is a downstream target of PLEK2. By reviewing the available literature, we found that LCN2 is an oncogene that has been comprehensively studied. Also known as siderocalin, uterocalin, NGAL [31], and oncogene 24p3, LCN2 is a secreted glycoprotein of the adipokine superfamily. LCN2 exists as a ~25 kDa monomer, a disulfide-linked homodimer, and a disulfide-linked heterodimer with matrix metalloproteinase 9 (MMP-9, gelatinase-B) [32–35]. Moreover, the relatively poorer prognosis of several aggressive forms of breast cancer [36], pancreatic cancer [37], and endometrial carcinoma is closely associated with overexpression of LCN2. For instance, increased levels of LCN2 promote not only proliferation but also angiogenesis of breast cancer cells [38, 39]. Studies have also indicated the tight relationship between high expression of LCN2 and EMT, invasion [40–42], and metastasis [36] in several types of tumours. The function of LCN2 in ESCC has been broadly examined. LCN2 is overexpressed and hypomethylated in ESCC and regulates the migration and invasion of ESCC through several pathways. LCN2 is recognized to be significantly correlated with cell differentiation and tumour invasion in ESCC [43]. There is literature that LCN2 promotes migration and invasion through the ERK1/2 pathway [44]. However, we did not detect a change in the ERK1/2 pathway when PLEK2 was stably knocked down. In contrast, we detected the activation of the Akt pathway either in the condition of high expression of PLEK2 or high expression of LCN2. These data are consistent with the results of Lee et al. who found that a high level of LCN2 results in inactivation of the c-Jun N-terminal kinase (JNK) and phosphatidylinositol 3’-kinase (PI3K)/Akt signalling pathways [45]. To demonstrate that LCN2 also has significance in aspect of clinical value, LCN2 and its receptor were recognized to be independent prognostic factors in ESCC [46]. However, we did not confirm the same conclusion after analysing TCGA database, perhaps because it is associated with the small number of paired samples in TCGA database.

## Conclusion

Our study adds to the accumulating evidence that suggests that PLEK2 may serve as a subtype-specific prognostic biomarker and a potential target for the treatment of ESCC. Additional studies are needed to confirm the underlying mechanism of the interaction between PLEK2 and LCN2.

## Supplementary information

Supplementary figures and tables
