# Supplementary material for: TGF-β-induced PLEK2 promotes metastasis and chemoresistance in oesophageal squamous cell carcinoma by regulating LCN2
Source: Cell Death Dis. 2021 Oct 2;12(10):901. doi: 10.1038/s41419-021-04155-z (PMC8487427; doi:10.1038/s41419-021-04155-z)
Supplement: Supplementary file 1 — Supplementary figures and tables [file 41419_2021_4155_MOESM1_ESM.docx]

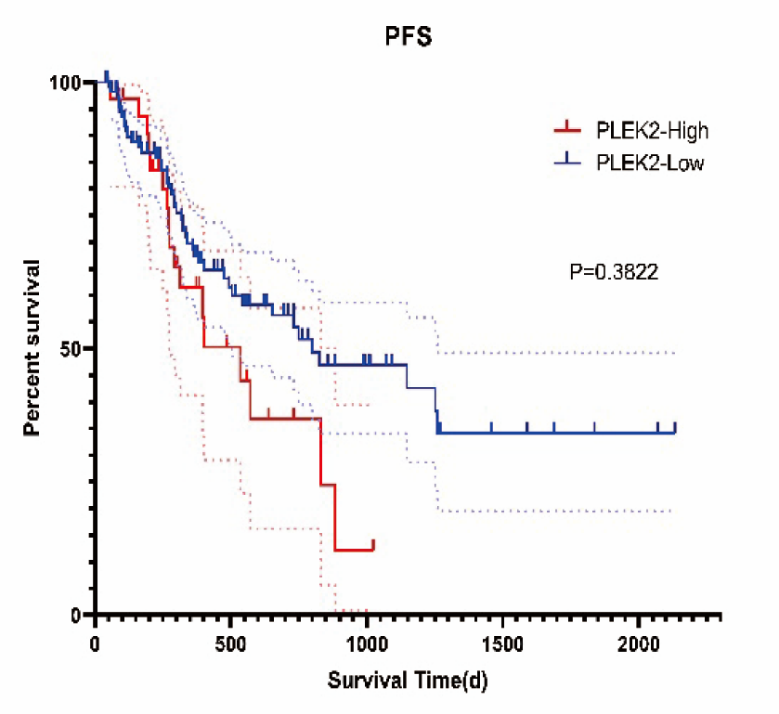


**Supplementary Figure S1**

Kaplan–Meier plots of OS of patients with ESCC in TCGA database, *P*=0.3822;


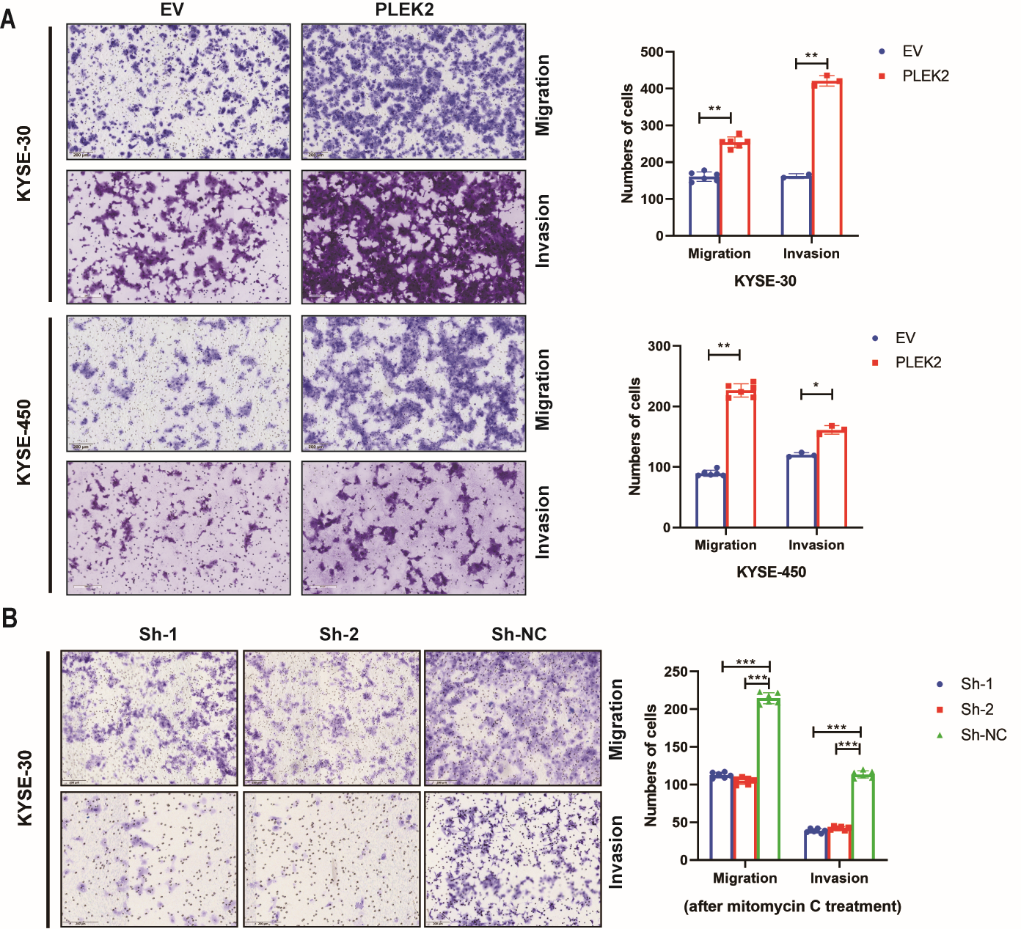


**Supplementary Figure S2**

(A)The migration and invasion ability of PLEK2 overexpression cells;

(B) The migration and invasion ability of PLEK2 knockdown cells after treatment of mitomycin C;


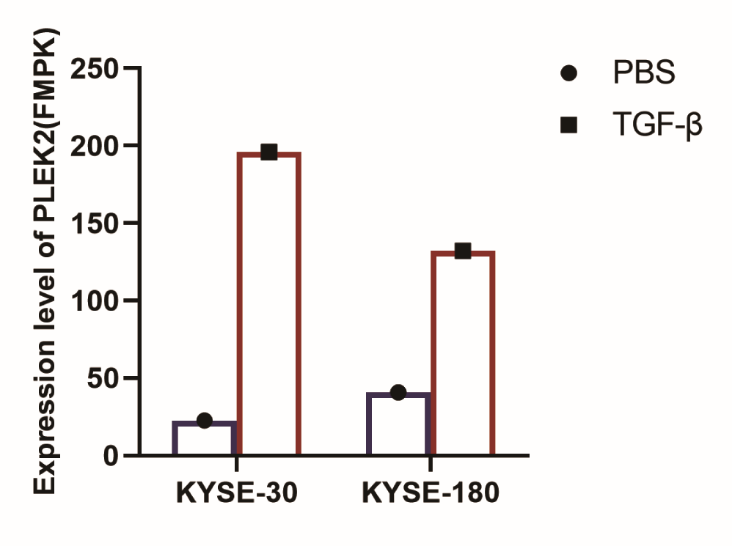


**Supplementary Figure S3**

RNA-seq result of ESCC cells whose PLEK2 expression chanced following TGF-β


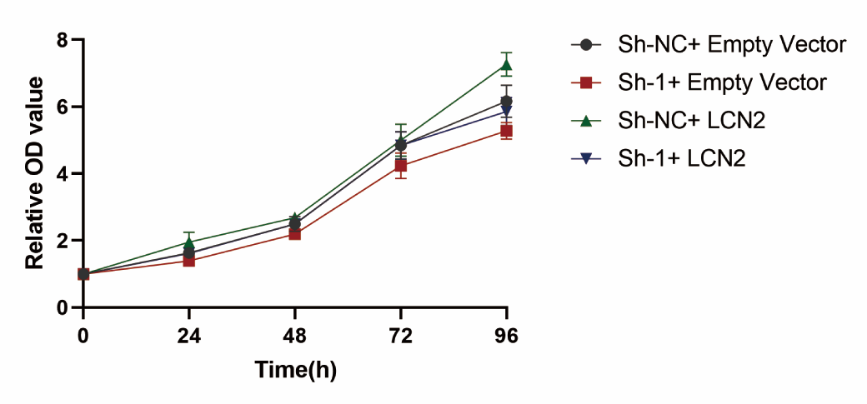


**Supplementary Figure S4**

The proliferation ability of PLEK2 knockdown cells with or without LCN2 overexpression.


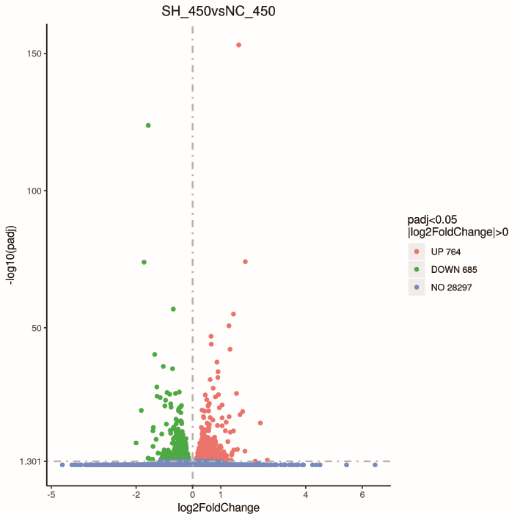


**Supplementary Figure S5**

Volcano plot of differential genes expression after PLEK2 was stably knockdown in KYSE-450 cells.

Supplementary Table 1  Characteristics of patients chosen to be used in microarray

| Characteristics | Training cohort  (N=100) |
| --- | --- |
| Gender, No. (%)  Male  Female | 66(66.0%)  34(34.0%) |
| Age, No. (%)  ≤60  >60 | 44(44.0%)  56(56.0%) |
| Subtype, No. (%)  LN+  LN- | 36(36.0%)  54(54.0%) |
| TNM Stage, No. (%)  1-2  3-4 | 68(68.0%)  32(32.0%) |
| OS state, No. (%)  Alive  Dead | 42(42.0%)  58(58.0%) |
| Location, No. (%)  Upper  Middle  Lower | 29(29.0%)  63(63.0%)  8(8.0%) |

Supplementary Table 2 Characteristics of patients enrolled in GSE53625

| Characteristics | Training cohort  (N=119) |
| --- | --- |
| Gender, No. (%)  Male  Female | 98(82.4%)  21(17.6%) |
| Age, No. (%)  ≤60  >60 | 61(51.3%)  58(48.7%) |
| Subtype, No. (%)  LN+  LN- | 65(54.6%)  54(45.45) |
| TNM Stage, No. (%)  1-2  3-4 | 53(44.5%)  66(55.5%) |
| OS state, No. (%)  Alive  Dead | 46(38.7%)  73(61.3%) |
| Location, No. (%)  Upper  Middle  Lower | 46(38.7%)  73(61.3%) |

Supplementary Table 3 Primers sequences of genes detected by qRT-PCR

| Genes | Forward (5’ to 3’) | Reverse (5’ to 3’) |
| --- | --- | --- |
| GAPDH | GGACGAGATCCCTCCAAAAT | GGCTGTTGTCATACTTCTCATGG |
| PLEK2 | GGAGTTAAGTGGCACGGTGGTG | CTTCCAGAGCAGACAVGAGTGAAC |
| LCN2 | GAGTTACCCTGGATTAACGAGT | AAGCGGATGAAGTTCTCCTTTA |

Supplementary Table 4 The predicted sequences of binding motifs of Smad2/3

| Matrix ID | Name | Score | Relative score | Start | End | Strand | Predicted sequence |
| --- | --- | --- | --- | --- | --- | --- | --- |
| MA0513.1 | SMAD2::SMAD3::SMAD4 | 11.2585 | 0.86862501 | 395 | 407 | - | CAGTCTCTCTGCT |
| MA0513.1 | SMAD2::SMAD3::SMAD4 | 8.14135 | 0.81775086 | 158 | 170 | + | GTGCCTGCCGCCT |

Supplementary Table 5 Primer sequences specific for the promoter region of PLEK2

| Primers | Forward (5’ to 3’) | Reverse (5’ to 3’) |
| --- | --- | --- |
| Sequences | taagcgttcctcgagggcaatg | gtggttggtcctccaggtacaga |
